# Regulation of *MYB* by distal enhancer elements in human myeloid leukemia

**DOI:** 10.1038/s41419-021-03515-z

**Published:** 2021-02-26

**Authors:** Mengjia Li, Penglei Jiang, Kai Cheng, Zehui Zhang, Shuyu Lan, Xiaoxia Li, Lirong Zhao, Yucheng Wang, Xiang Wang, Jing Chen, Tao Ji, Bingshe Han, Junfang Zhang

**Affiliations:** 1 Key Laboratory of Exploration and Utilization of Aquatic Genetic Resources, Ministry of Education, Shanghai Ocean University, Shanghai, China; 2 National Demonstration Center for Experimental Fisheries Science Education, Shanghai Ocean University, Shanghai, China; 3 International Research Center for Marine Biosciences, Ministry of Science and Technology, Shanghai Ocean University, Shanghai, China; 4 Department of Hematology/Oncology, Shanghai Children’s Medical Center (SCMC), Shanghai Jiao Tong University School of Medicine, Shanghai, China

**Keywords:** Oncogenes, Chromatin structure

## Abstract

MYB plays vital roles in regulating proliferation and differentiation of hematopoietic progenitor cells, dysregulation of MYB has been implicated in the pathogenesis of leukemia. Although the transcription of *MYB* has been well studied, its detailed underlying regulatory mechanisms still remain elusive. Here, we detected the long-range interaction between the upstream regions, −34k and −88k, and the *MYB* promoter in K562, U937, and HL-60 cells using circularized chromosome conformation capture (4C) assay, which declined when *MYB* was downregulated during chemical-induced differentiation. The enrichment of enhancer markers, H3K4me1 and H3K27ac, and enhancer activity at the −34k and −88k regions were confirmed by ChIP-qPCR and luciferase assay respectively. ChIP-qPCR showed the dynamic binding of GATA1, TAL1, and CCAAT/enhancer-binding protein (C/EBPβ) at −34k and −88k during differentiation of K562 cells. Epigenome editing by a CRISPR-Cas9-based method showed that H3K27ac at −34k enhanced TF binding and *MYB* expression, while DNA methylation inhibited *MYB* expression. Taken together, our data revealed that enhancer elements at −34k are required for *MYB* expression, TF binding, and epigenetic modification are closely involved in this process in human myeloid leukemia cells.

## Introduction

The transcription factor MYB is a key regulator for hematopoiesis^1,2^. Dysregulation of MYB often associates with various hematological disorders including acute myeloid leukemia (AML), chronic myeloid leukemia (CML), and acute lymphoblastic leukemia (ALL)^3–5^. Aberrant expression of MYB has been also reported in malignant solid tumors including colon cancer, breast cancer, adenoid cystic carcinoma, and brain cancer^6–9^. Recurrent chromosomal translocation, genomic duplication, C-terminal truncation, and N-terminal truncation contribute to *MYB* have been reported in human leukemia^10–13^.

The expression of MYB is precisely regulated under physiological conditions. Previous studies indicated that *MYB* transcription is mainly regulated through a transcriptional attenuation site within the first intron^14,15^. miRNAs including miR-150 and miR-17-92 can target *MYB* mRNA in a stage-specific manner^16,17^. PU.1 negatively regulates the *c-myb* promoter during granulocytic differentiation^18^. *MYB* is also an essential downstream target of Hoxa9/Meis1 in hematopoietic cells^19^.

Recently increasing studies support that distal regulatory elements play vital roles in *MYB* regulation. Transgene insertion 77 kb upstream of *c-myb* markedly decreases *c-myb* expression in mouse^20^. Multiple distal regions 36, 61, 68, 81, and 109 kb upstream of *c-myb* are involved in *c-myb* regulation in erythroid differentiation in mouse^21^. Retroviral insertions upstream and downstream of *c-myb* lead to upregulation of *c-myb* in murine and feline cell lines^22,23^. And our previous study identified three murine leukemia virus integration regions (located at −25k, −56k, and −70k), which interact with *c-myb* through DNA looping and facilitate the integrated virus to activate *c-myb* expression in murine myeloid progenitor M1 cells^23,24^. We further demonstrated that the regulatory element at the −28k region has an essential role in *c-myb* regulation during IL-6 induced differentiation in M1 cells^25^. Distal enhancer elements have been also identified upstream and downstream of *MYB* in human. The −84k and −71k regions of *MYB* can regulate *MYB* and fetal hemoglobin in primary human erythroid progenitors (HEPs)^26^. An enhancer ~140 kb downstream of *MYB* was recently identified with improved experimental and computational parameters from single-cell enhancer screens^27^. Above observations support multiple distal elements regulate *MYB* in a cell-type-specific manner, however the detailed mechanisms of distal elements in *MYB* regulation in human leukemia cells remain to be elucidated.

Here, we showed the association of the −34k and −88k regions of *MYB* and the *MYB* promoter in human leukemia cells using circularized chromosome conformation capture (4C) assay. And these regions are enriched for enhancer characteristics including hallmark histone modifications, TF binding and enhancer activity in luciferase assay. Further study showed the dynamic DNA looping formation, TF binding, and epigenetic modifications at the −34k region are involved in *MYB* expression during differentiation of human myeloid leukemia cells.

## Results

### Long-range interaction between the *MYB* promoter and its upstream distal regions in human myeloid leukemia cells

**Fig. 1:**
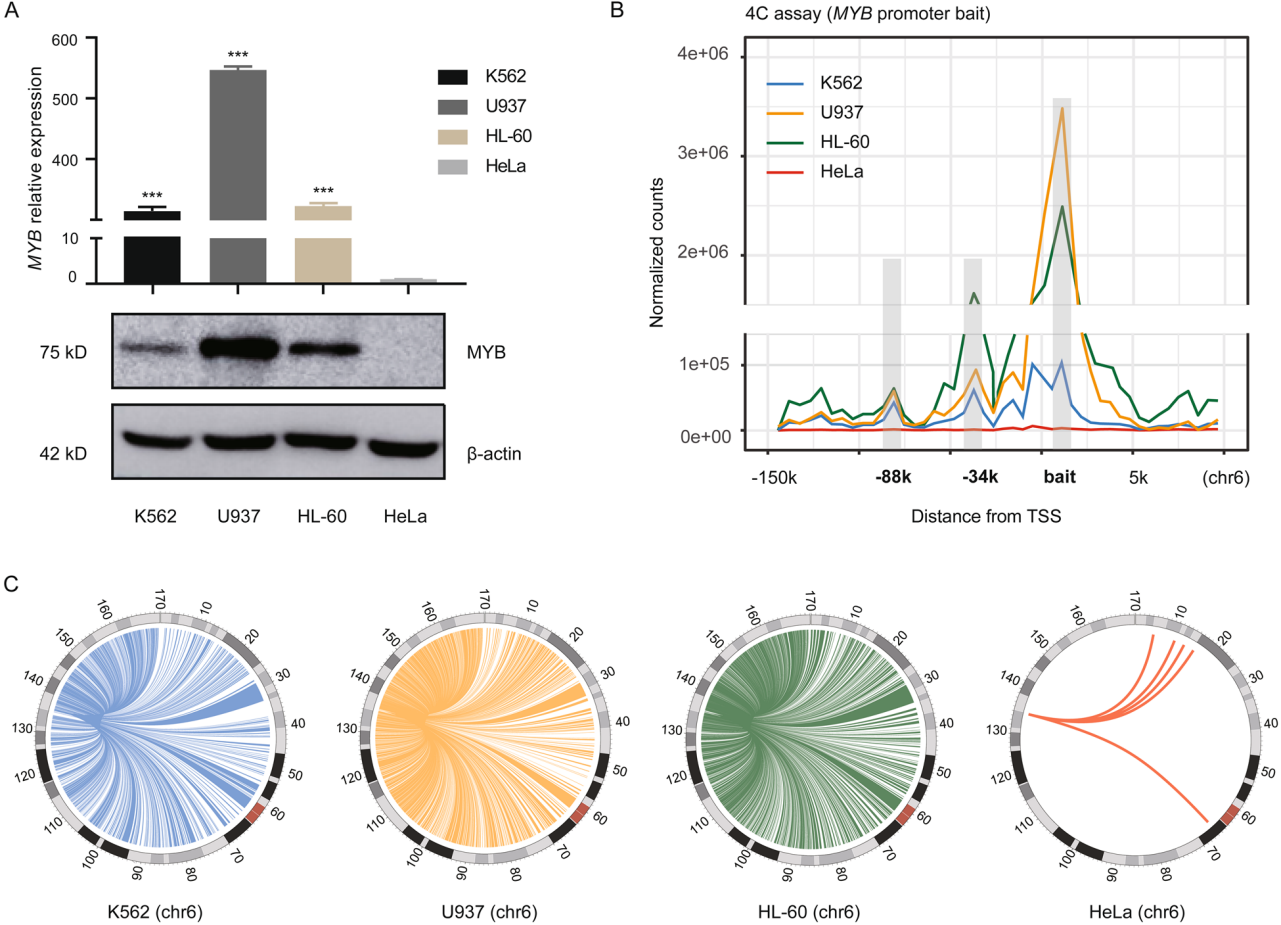
Long-range interaction between the *MYB* promoter and its upstream distal regions in human myeloid leukemia cells. **A** MYB mRNA (upper) and protein (lower) levels were determined in K562, U937, HL-60, and HeLa cell lines, using RT-qPCR and Western blot respectively. *Gapdh* was used as an internal control, β-actin was used as a loading control. Data are represented as mean ± SD of three independent experiments, and *P* values are calculated using Student’s *t*-test (*P < 0.05; **P < 0.01; ***P < 0.001). **B** 4C assay was performed with the *MYB* promoter as viewpoint in indicated cells. The diagram shows the near-bait interactions on chromosome 6 in indicated cells, the distribution plot of normalized read counts was generated by the 4C-ker to visualize proximity of the peaks to the bait. Upstream regions showing high frequency of interaction are highlighted. Distance of indicated regions from transcription start site (TSS) is shown. **C** Circos diagrams depicting the 4C-seq contact map for intra-chromosomal interactions in indicated cell lines called by 4C-ker.

To investigate the distal regulatory elements interacting with the *MYB* promoter, 4C assay was performed in human myeloid leukemia cells. K562, U937, and HL-60 cells all expressed high levels of MYB, while MYB was not detected in HeLa cells (Fig. 1A), the result is consistent with previous studies^28,29^. Using the *MYB* promoter as the bait fragment, 4C assay detected strong near-bait interactions on chromosome 6 in all three leukemia cell lines (K562, U937, and HL-60) except for HeLa cells, among which two prominent peaks of interactions were detected around −34k and −88k upstream of the *MYB* gene (Fig. 1B). The peaks located at the −34k and −88k regions indicate the physical interaction of these regions with the *MYB* promoter.

We further scanned the potential regulatory elements involved in intra-chromosomal interaction with the *MYB* promoter, and a Circos diagram was generated to show the 4C-seq contact map for *cis* interactions on chromosome 6 in all four cell lines (Fig. 1C). A dramatic difference in the abundance of long-range contacts along chromosome 6 was observed between leukemia cells and HeLa cells, much more *MYB* related intra-chromosomal interactions were detected in leukemia cells than in HeLa cells. Meanwhile, our data also showed the potential regulatory elements interacting with the *MYB* promoter at the genome-wide level, which showed a high interaction frequency based on the top 100 regions (supplementary Table 2). These observations suggest that multiple distal regions, especially those within the *HBS1L-MYB* intergenic region, interact with the *MYB* promoter via long-range DNA interaction.

### The −34k and −88k regions of *MYB* are enriched for enhancer features

**Fig. 2:**
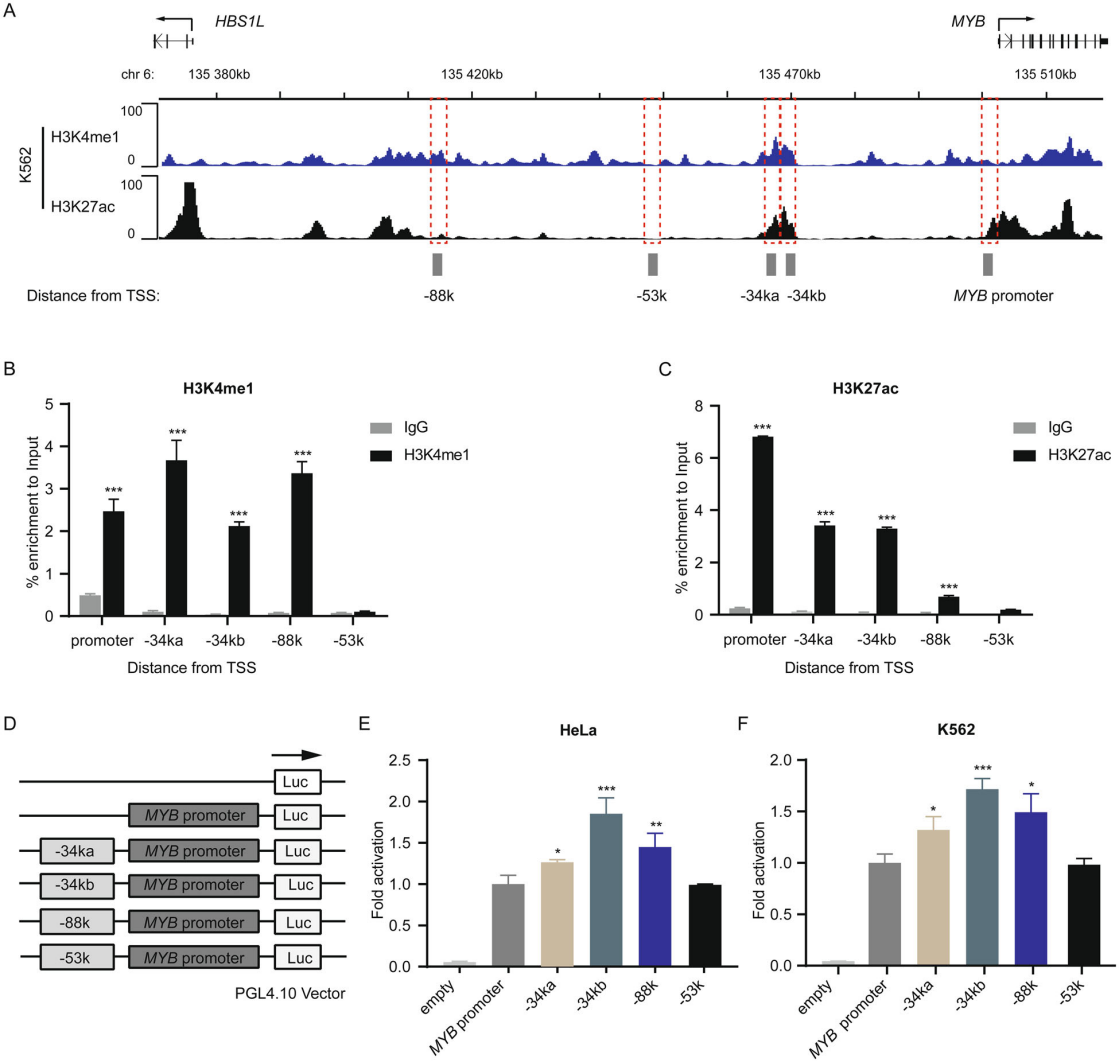
The −34k and −88k regions of *MYB* are enriched for enhancer features. **A** Public ChIP-seq data show H3K4me1 and H3K27ac profiles in the *HBS1L-MYB* region, the *MYB* promoter interaction regions −34ka, −34kb, and −88k are enriched for H3K4me1 and H3K27ac (−53k, negative control). The *y* axis represents sequence tag density. Genome-wide data sets were obtained from the ENCODE consortium and accessed through the WashU Epigenome Browser. **B**, **C** ChIP-qPCR was performed to detect the enrichment of H3K4me1 (**B**) and H3K27ac (**C**) at indicated regions in K562 cells. Values are represented as percent input normalized by immunoglobulin G control. **D** A schematic view of the constructs for enhancer activity assay. **E**, **F** Dual-luciferase reporter assay was performed to show the enhancer activity of indicated distal regions in HeLa (**E**) and K562 (**F**) cells. The pGL4.10-*MYB*-promoter was used as control. Luciferase signals were normalized to renilla signals. Data are represented as mean ± SD of three independent experiments, and *P* values are calculated using Student’s *t*-test (*P < 0.05; **P < 0.01; ***P < 0.001) in **B**, **C**, **E**, and **F**.

H3K4me1 and H3K27ac are two commonly used hallmarks to identify putative genome-wide enhancers^30,31^ (Fig. 2A). ChIP-seq data from ENCODE (Encyclopedia of DNA Elements) show strong enrichment of H3K4me1 and H3K27ac at −34k, while strong enrichment of only H3K4me1 was detected at −88k. The enrichment of H3K4me1 (Fig. 2B) and H3K27ac (Fig. 2C) at −34k and −88k was further confirmed using ChIP-qPCR. The DNA fragments representing H3K4me1 peaks, named −34ka (1034 bp), −34kb (1055 bp), and −88k (1388 bp), respectively (Fig. 2A), were cloned and inserted upstream of the *MYB* promoter controlling a firefly luciferase reporter gene (Fig. 2D), a −53k fragment without H3K4me1 enrichment was used as control. The constructs were transfected into HeLa (Fig. 2E) and K562 cells (Fig. 2F), respectively. Compared with the −53k fragment, the −34ka, −34kb, and −88k fragments all showed significantly increased luciferase activity (Fig. 2E, F). The −34kb fragment showed the highest enhancer activity. Taken together, these data indicate that these distal regions contain enhancers for *MYB* transcription.

### Dynamic long-range interaction of the *MYB* locus during differentiation of human leukemia cells

**Fig. 3:**
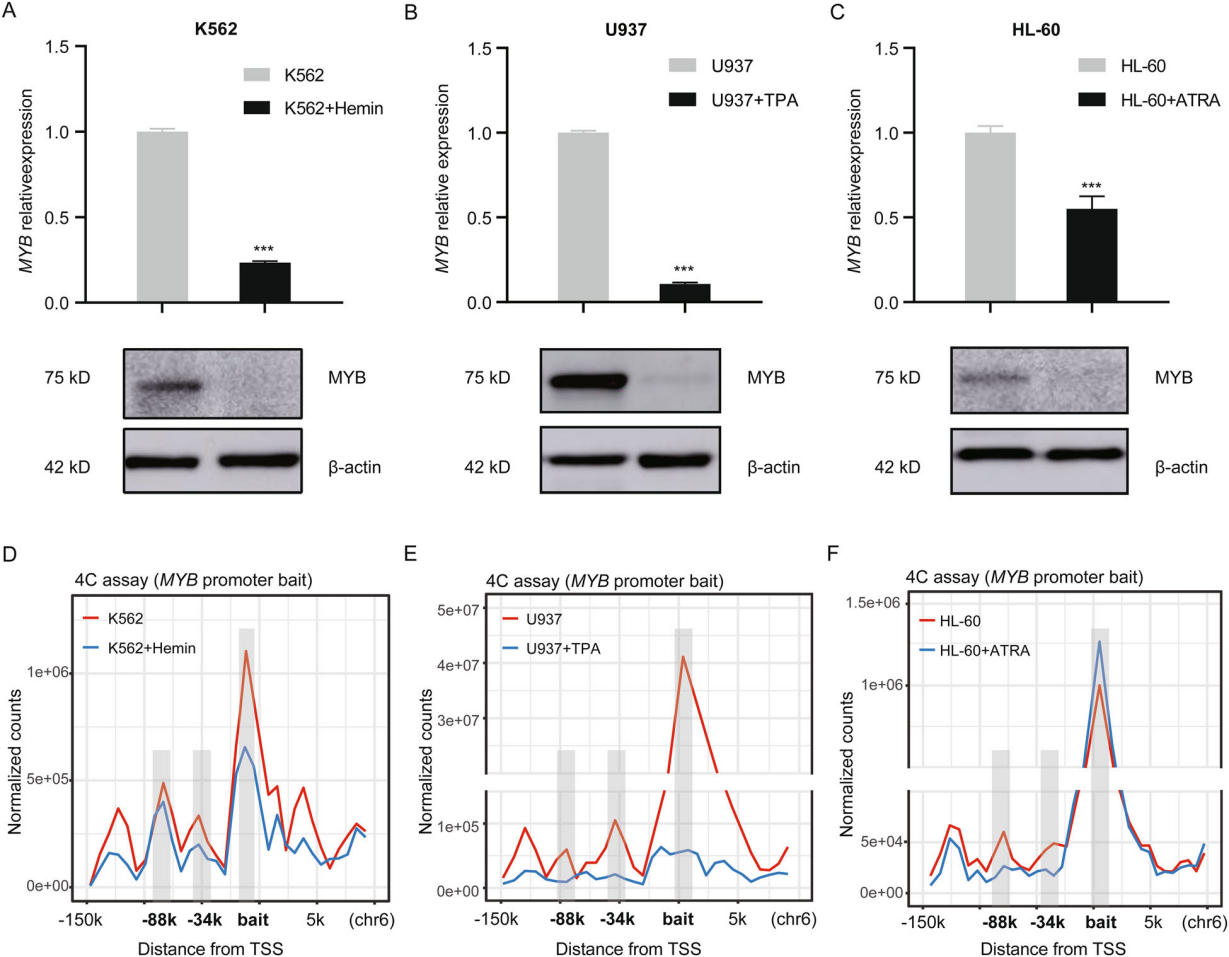
Dynamic long-range interaction of the *MYB* locus during differentiation of human leukemia cells. K562, U937, and HL-60 cells were treated with hemin, TPA and ATRA for erythroid, monocytic, and granulocytic differentiation, respectively. Then **A**–**C** MYB mRNA (upper) and protein (lower) levels were determined in K562 (**A**), U937 (**B**), and HL-60 (**C**) cells, using RT-qPCR and Western blot respectively. *Gapdh* was used as an internal control, β-actin was used as a loading control. Data are represented as mean ± SD of three independent experiments, and *P* values are calculated using Student’s *t*-test (*P < 0.05; **P < 0.01; ***P < 0.001) in **A**–**C**. **D**–**F** 4C assay was performed with the *MYB* promoter as viewpoint in indicated cells. Diagrams show the change of near-bait interactions on chromosome 6 in K562 (**D**), U937 (**E**), and HL-60 (**F**) cells during differentiation, the distribution plots of normalized read counts were generated by the 4C-ker to visualize proximity of the peaks to the bait. Interacting upstream regions of the *MYB* locus were highlighted. Distance of marked regions from transcription start site (TSS) is shown.

We treated K562, U937 and HL-60 cells with hemin, 12-O-Tetradecanoylphorbol 13-acetate (TPA) or all-trans-retinoic acid (ATRA) to induce erythroid, monocytic and granulocytic differentiation, respectively. *MYB* mRNA and protein levels reduced remarkably after treatment in all three cell lines (Fig. 3A–C), which is consistent with previous studies that *MYB* is highly expressed in immature proliferating haematopoietic cells, and strongly downregulated during terminally differentiation^32–34^. 4C assay was subsequently carried out using the *MYB* promoter as the bait fragment (Fig. 3D–F). In untreated cells high frequency long-range interaction between the *MYB* promoter and distal regions was observed, however the frequency of most long-range interactions strikingly diminished upon differentiation, especially at the −34k and −88k regions. Meanwhile, the intra-chromosomal interactions with the *MYB* promoter significantly altered during differentiation in all tested cell lines (Supplemental Fig. 1A–C). The potential inter-chromosomal interaction with the *MYB* promoter also showed dramatic changes during differentiation in all tested cell lines (Supplementary Tables 3–5). This finding is consistent with previous study that gene-regulatory chromatin interactions were altered upon ATRA induction in HL-60 cells^35^. Thus, we concluded that downregulation of *MYB* upon differentiation is accompanied by a loss of communication between the *MYB* promoter and above distal enhancers.

### Binding of transcription factors at distal enhancers of *MYB* during differentiation

**Fig. 4:**
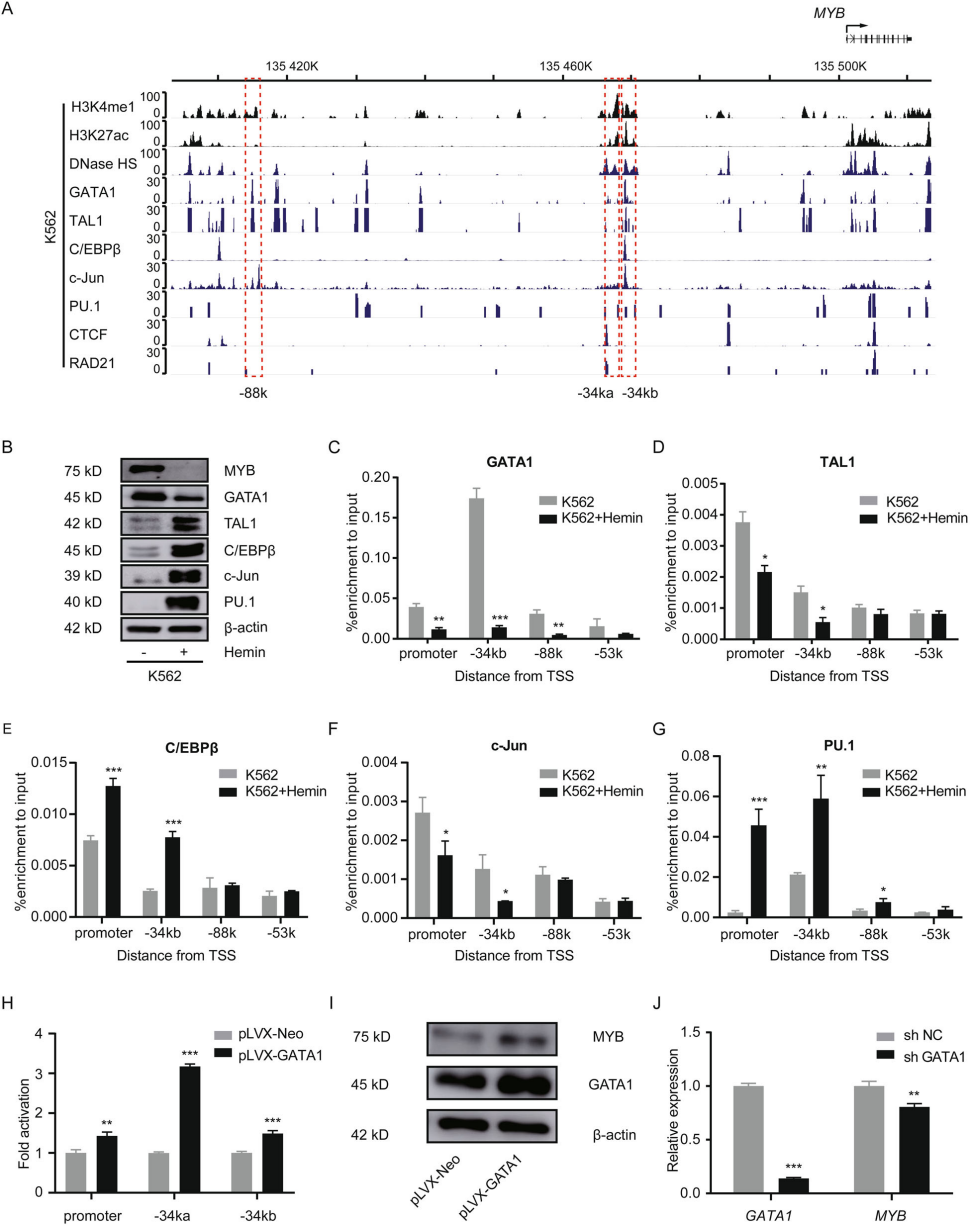
Binding of transcription factors at distal enhancers of *MYB* during differentiation. **A** Public ChIP-seq data were analyzed to show profiles of indicated histone modifications and transcription factors in the *HBS1L-MYB* region in K562 cells. The *y* axis represents sequence tag density. Genome-wide datasets were obtained from the ENCODE consortium and accessed through the WashU Epigenome Browser. **B** K562 cells were treated with 30 µM hemin for 72 h, the expression of indicated transcription factors was detected using Western blot. β-actin was used as a loading control. **C**–**G** K562 cells were treated with hemin, then the binding of GATA1 (**C**), TAL1 (**D**) C/EBPβ (**E**), c-Jun (**F**), and PU.1 (**G**) was detected with ChIP-qPCR at promotor, −34kb and −88k regions. **H** pGL4.10-*MYB*-promter reporter constructs containing the −34ka or −34kb region were transfected into 293T cells with control or GATA1 overexpression, then luciferase activity was determined. Luciferase signals were normalized to renilla signals. **I** K562 cells were infected with GATA1 overexpressing lentivirus, 72 h after viral infection, MYB and GATA1 expression was detected with Western blot, β-actin was used as a loading control. **J** K562 cells were infected with lentiviral particles expressing GATA1-shRNA for 72 h, then expression *GATA1* and *MYB* was detected with RT-qPCR. *Gapdh* was used as an internal control. Data are represented as mean ± SD of three independent experiments, and *P* values are calculated using Student’s *t*-test (*P < 0.05; **P < 0.01; ***P < 0.001) in **C**–**H**, **J**.

We further investigated the roles of TFs at above enhancers in *MYB* regulation. Public ChIP-seq data of histone marks, DNase I hypersensitivity (DNase HS) and TF profiles were generated by the ENCODE project in the *HBS1L-MYB* region. DNase HS and the enrichment of GATA1, TAL1, C/EBPβ, c-Jun, and PU.1 were observed at the −34ka and/or −34kb regions (Fig. 4A). The enrichment of GATA1, TAL1, and c-Jun were observed at the −88k region (Fig. 4A). Strong enrichment of CTCF and Rad21, which participate in long-range chromatin interactions in the vicinity of 4C interaction sites, was observed mainly near the −34k region (Fig. 4A). The enrichment of GATA1, TAL1, and C/EBPβ was further confirmed by ChIP-qPCR in K562 cells (Supplemental Fig. 2A–C).

We investigated the binding of selected TFs at −34k and −88k regions during differentiation. Western blot showed that GATA1 decreased significantly, while TAL1, C/EBPβ, c-Jun, and PU.1 increased during hemin-induced differentiation in K562 cells (Fig. 4B). After hemin treatment, the binding of GATA1, TAL1, c-Jun decreased at −34kb, PU.1, and C/EBPβ binding increased at −34kb and promoter; while the GATA1 binding decreased and PU.1 binding increased at −88k (Fig. 4C–G).

To further assess the effect of TF binding to these distal regions, we determined the luciferase activity of the −34k enhancer constructs after GATA1 overexpression in 293T cells (Fig. 4H). Unexpectedly, a 3-fold increase of luciferase activity of the −34ka construct was observed after GATA1 overexpression, while only a moderate increase was observed for luciferase activity of the −34kb construct and the construct contains only the *MYB* promoter, indicating that GATA1 binding is required for enhancer activity of the −34k region. Meanwhile, we overexpressed and knocked down GATA1 in K562 cells via lentiviral transduction. Our results showed that overexpression of GATA1 increased MYB expression (Fig. 4I), while GATA1 knockdown reduced *MYB* expression (Fig. 4J), indicating GATA1 plays an important role in *MYB* expression, and corroborating a previous study that GATA1 overexpression leads to failure to repress *MYB* during erythroid differentiation of K562 cells^36^. Together, our data showed that TF binding at the −34k enhancer elements play a critical role in *MYB* expression in K562 cells.

### Epigenetic modification of distal enhancers affects *MYB* expression

**Fig. 5:**
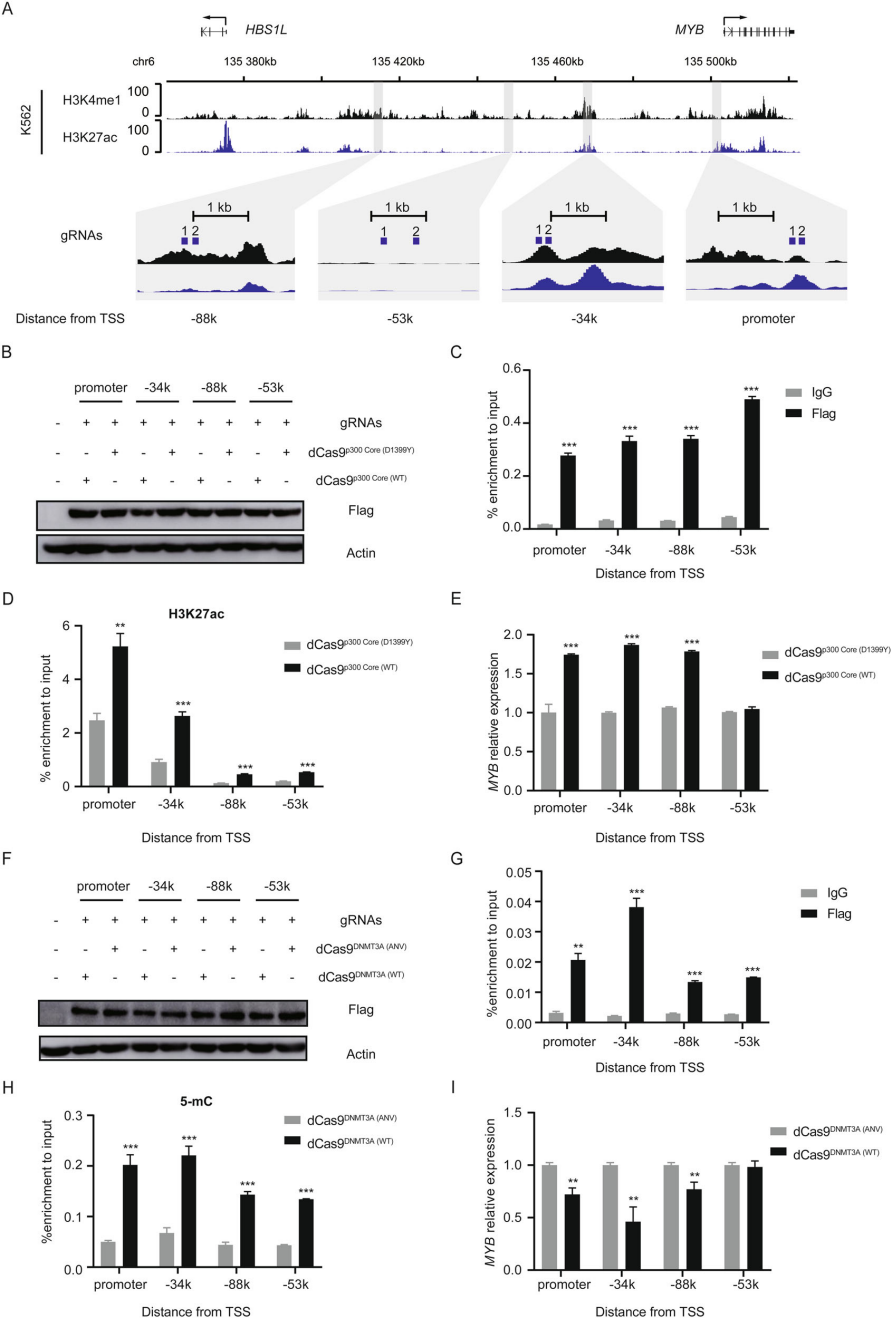
Epigenetic modification of distal enhancers affects *MYB* expression. **A** The dCas9^p300 Core^ and dCas9^DNMT3A^ fusion proteins modify chromatin at a targeted enhancer. The region encompassing the *HBS1L-MYB* intergenic region on chromosome 6 (135,376,037–135,502,452; GRCh37/hg19 assembly) is shown. gRNA target locations are indicated in blue with corresponding black numbers. ENCODE/Broad Institute H3K4me1 and H3K27ac enrichment signal in K562 cells is shown for comparison. Magnified insets for the *MYB* enhancer and promoter regions are displayed below. **B**–**E** K562 cells were co-transfected with indicated dCas9^p300 Core^, and gRNAs targeted to the each *MYB* promoter and enhancer region. Then expression levels of dCas9^p300 Core^ fusion protein were detected with Western blot (**B**); dCas9p^300 Core^ fusion protein (**C**) and H3K27ac (**D**) enrichment at indicated regions was detected using ChIP-qPCR, and *MYB* mRNA levels were determined using RT-qPCR (**E**). dCas9^p300 Core (D1399Y)^ contained a nonfunctional residue substitution at the acetyltransferase domain and was used as a negative control. **F**–**I** K562 cells were co-transfected with indicated dCas9^DNMT3A^ fusion protein, and gRNAs targeted to the each *MYB* promoter enhancer region. Then expression levels of dCas9^DNMT3A^ fusion protein were detected with Western blot (**F**); dCas9^DNMT3A^ fusion protein (**G**) and 5-methylcytosine (**H**) enrichment at indicated regions was detected using ChIP-qPCR, and *MYB* mRNA levels were determined using RT-qPCR (**I**). dCas9^DNMT3A (ANV)^ contained a nonfunctional residue substitution at the DNA methylation domain and was used as a negative control. Data are represented as mean ± SD of three independent experiments, and *P* values are calculated using Student’s t-test (*P < 0.05; **P < 0.01; ***P < 0.001) in (**C**–**E**, **G**–**I**).

Epigenetic modification can affect enhancer activity, probably via regulation of chromatin structure and TF binding^37^. To test the effect of epigenetic modification on the function of above enhancers, here we applied a dCas9-based epigenome editing method^38,39^. After co-transfection into K562 cells, a dCas9^p300 Core^ fusion protein with a Flag tag was co-expressed with 2 gRNAs^38^, which target one specific site, then *MYB* expression was examined (Fig. 5A). As shown in Fig. 5B, dCas9^p300 Core^ was expressed in K562 cells after transfection. ChIP-qPCR using an antibody against the Flag epitope showed that dCas9^p300 Core^ was recruited to the targeted sites by the gRNAs (Fig. 5C) and upregulated H3K27ac level at these sites specifically (Fig. 5D). And *MYB* expression was increased by elevated H3K27ac at the promoter, −34k and −88k regions (Fig. 5E), indicating that H3K27ac at these sites upregulates *MYB* expression.

Meanwhile, the effect of local DNA methylation at these sites on *MYB* transcription was also investigated using a previously reported dCas9^DNMT3A^ fusion protein^40^. Figure 5F showed that dCas9^DNMT3A^ was expressed in K562 cells after transfection. ChIP-qPCR using a Flag epitope antibody showed that dCas9^DNMT3A^ was recruited to the targeted sites by the gRNAs (Fig. 5G), and upregulated 5-methylcytosine (5-mC) level at these sites specifically (Fig. 5H). And *MYB* expression was decreased by elevated 5-mC level at the promoter, −34k and −88k regions (Fig. 5I), indicating that DNA methylation can inhibit the function of these DNA elements.

### Epigenetic modification of distal enhancers affects TFs binding and cell differentiation

**Fig. 6:**
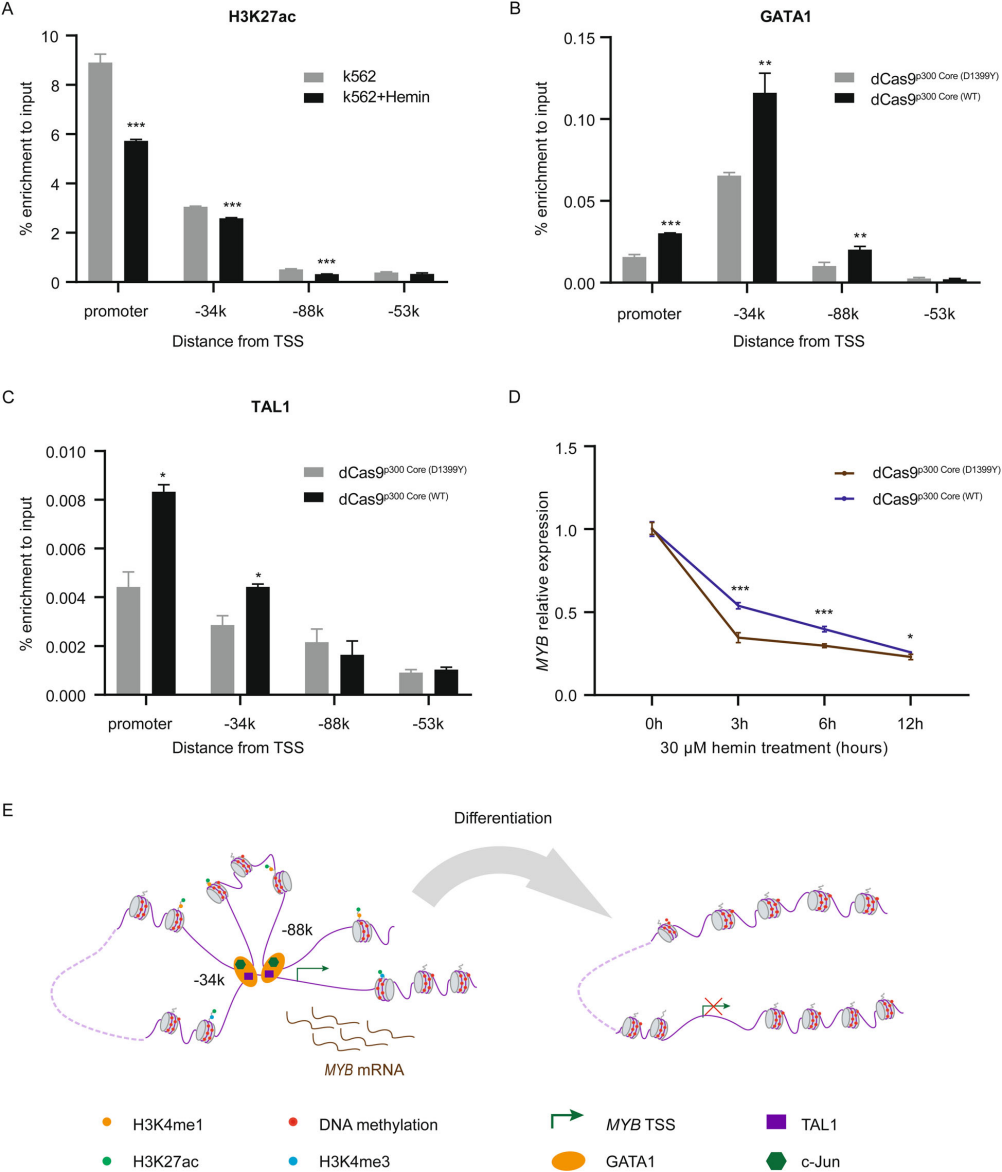
Epigenetic modification of distal enhancers affects TFs binding and cell differentiation. **A** H3K27ac ChIP-qPCR enrichment at the *MYB* promoter and enhancers in K562 cells treated with hemin to induce erythroid differentiation. **B**, **C** GATA1 (**B**) and TAL1 (**C**) ChIP-qPCR enrichment at the *MYB* promoter and enhancers in K562 cells co-transfected with the indicated dCas9^p300 core^ and two gRNAs targeted to the each *MYB* promoter and enhancer region. dCas9^p300 Core (D1399Y)^ contained a nonfunctional residue substitution at the acetyltransferase domain and was used as a negative control. **D** K562 cells co-transfected with the indicated dCas9^p300 Core^ fusion protein and two gRNAs targeted to the −34k region were treated with hemin for indicated times, then *MYB* mRNA levels were determined using RT-qPCR. **E** Model of the dynamic long-range interaction during differentiating of human leukemia K562 cells. Distal cis-regulatory elements (−34k and −88k regions) enriched for activating epigenetic modification H3K4me1 and H3K27ac, and transcription factors containing GATA1 (orange ovals), TAL1 (violet rectangles) and c-Jun (green diamonds), allowing for high-level expression of *MYB*. During differentiation, intergenic transcription factor occupancy decreases at the cis-regulatory elements, leading to a destabilization of the DNA-looping and a dramatic decrease of *MYB* transcription, allowing cells to terminally differentiate. Data are represented as mean ± SD of three independent experiments, and *P* values are calculated using Student’s *t*-test (*P < 0.05; **P < 0.01; ***P < 0.001) in **A**–**D**.

To further understand the roles of epigenetic modification at above sites in *MYB* regulation, we examined the effect of H3K27ac on TF binding and cell differentiation in K562 cells. ChIP-qPCR showed that H3K27ac enrichment significantly reduced at the *MYB* promoter, −34k and −88k enhancer regions during hemin induced differentiation (Fig. 6A). dCas9^p300 Core^ targeting enhanced GATA1 binding at promoter, −34k and −88k (Fig. 6B), while dCas9^p300 Core^ targeting enhanced TAL1 binding at promoter and −34k (Fig. 6C), indicating that H3K27ac helped binding of these two TFs at above DNA elements. Furthermore, dCas9^p300 Core^ targeting at −34k could counter downregulation of *MYB* during early stages of hemin treatment (Fig. 6D), but failed to efficiently block hemin-induced repression of *MYB* eventually. As represented in our model (Fig. 6E), these data suggest that epigenetic modification at the *MYB* distal enhancers could affect TF binding and *MYB* expression during differentiation in K562 cells.

## Discussion

Mounting evidence indicates that the *MYB* gene is under regulation by distal enhancers. Multiple distal upstream and downstream regulatory elements have been reported from −25k ∼−250k regions of *c-myb* in mouse, which are involved in *c-myb* regulation in erythroid differentiation and leukemogenesis^20–25^. In human cells, distal enhancer elements have been identified at regions 84 kb, 71 kb upstream, and 140 kb downstream of *MYB*^26,27^. Above studies indicate that the *MYB* locus is under control of a complex regulatory network involving multiple upstream and downstream regulatory elements. However, how these enhancers coordinate in *MYB* regulation is unclear so far.

Using 4C assay showed that the −34k, −88k upstream regions interact with the *MYB* promoter, and binding sites of CTCF and Rad21 were identified near the −34k, −88k regions using public ChIP-seq data. The enhancer activity of the −34k and −88k regions was further confirmed by enrichment of H3K4me1 and H3K27ac and luciferase reporter assay (Figs. 1 and 2). Meanwhile, the binding of TFs (GATA1, TAL1, C/EBPβ, c-Jun, and PU.1) at −34k and −88k during differentiation was confirmed by ChIP-qPCR. Above data showed that the enhancer elements at the −34k, −88k regions interact with the *MYB* promoter in human K562, U937, and HL-60 leukemia cells. However, in human erythroid progenitors, long-range interactions with the *MYB* promoter were detected at multiple upstream sites ranging from −63k to −92k, which affect *MYB* expression^26^. Above evidence indicates that long-range interactions between distal enhancers and the *MYB* promoter may perform in a cell-specific manner.

By further analysis of the 4C-assay data, additional long-range interaction with the *MYB* promoter in K562, U937, and HL-60 cells was detected from more sites, including −107k and +140k regions (Supplementary Table 6), along chromosome 6 at much lower frequency than the −34k and −88k regions. Our results are consistent with a recent report that an enhancer ~140k downstream of *MYB* can regulate *MYB* expression in K562 cells via DNA-looping^27^, supporting that the enhancer at +140k is required for *MYB* regulation. Meanwhile, many other intrachromosome and interchromosome sites showed interaction with the *MYB* promoter in our analysis, the potential roles of these interactions are unknown so far.

Distal enhancers contribute to the activation of gene transcription via conformational loops that bring them physically close to gene promoters^41^. Lineage-specific dynamic and enhancer–promoter contacts cooperate in terminal differentiation^37^. We observed a loss of long-range interaction frequency from −34k and −88k during differentiation of human leukemia cells, accompanied by *MYB* downregulation. And a significant decline of long-range interaction from other intrachromosome and interchromosome sites was also observed. The loss of chromatin looping between distal enhancers and the *c-myb* promoter during differentiation was also reported in MEL cells^21^. Above data showed the dynamic long-range interaction between the *MYB* promoter and the −34/−88k regions during hemin induced differentiation in K562 cells.

TFs are required for enhancer function and involved in establishing and stabilizing long-range chromatin interactions^42^. We first showed that GATA1 binds to the −34k and −88k enhancer regions and upregulates *MYB* expression (Fig. 4). GATA1 is considered as the “master” transcription factor in erythropoiesis^43,44^. And it has been reported that GATA1 and TAL1 bind to the −71k and −84k enhancer regions of the *MYB* locus and positively regulate *MYB* expression along erythroid cell differentiation^26^. c-Jun binding at the promoter and −34k regions decreased during differentiation, our results support the idea that c-Jun principally binds to distal enhancers, and promoters and is considered a pioneer factor in modulating chromatin structure of distal enhancers in K562 cells^45^. PU.1 has been reported to suppress *MYB* expression through direct binding to the *MYB* promoter and recruitment corepressors HDAC1 and/or DNMT3a/b^46^. Here, we found that PU.1 binding at the promoter and −34k regions increased during differentiation, indicating that PU.1 in the *MYB* enhancer regions can also downregulate *MYB* expression. Furthermore, C/EBPβ binding at the promoter and −34k regions increased during differentiation, and the expression of C/EBPβ was indeed changed during myeloid differentiation. C/EBPβ acts as a transcription repressor for genes of liver proliferation^47^. Further studies will be required to determine whether C/EBPβ has a specific function in *MYB* control during myeloid differentiation.

Recent studies typically suggest that active enhancers display lower 5-mC levels than poised or silent enhancers, along with TF binding as well as the presence of active histone marks H3K4me1 and H3K27ac^48,49^. Here, we showed that the induction of histone acetylation at the −34k and −88k regions enhanced *MYB* transcription and TF binding (Figs. 5 and 6). Conversely, DNA methylation at the −34k and −88k regions leads to downregulation of *MYB*. Our results coincide with that hypermethylation at enhancers is generally associated with reduced chromatin accessibility and decreased TFs binding^50^. DNA methylation of enhancers can influence cell-type specific gene expression, and regulate relevant genes in acute myeloid leukemia and chronic myeloproliferative neoplasms^51^. And histone acetylation is required for enhancer function^52^. Cooperation of TFs with epigenetic modifications of chromatin contributes to the activation of regulatory elements including promoters and enhancers, TFs must gain access to their binding sites, and binding of TFs also modify the chromatin landscape^53^. Meanwhile, enhanced histone acetylation at the −34k region alone only delayed but failed to block hemin-induced repression of *MYB* (Fig. 6D), which is consistent with the relatively minor decrease in H3K27ac at this region upon hemin-induced differentiation (Fig. 6A), indicating that H3K27ac at this region is of secondary importance in hemin induced downregulation of *MYB*.

In the present study, we identified enhancer element at the −34k and −88k regions, which play roles in regulation of *MYB* in human leukemia cells. Our data will help understanding the mechanisms of regulation/dysregulation of *MYB* under physiological and pathological conditions in human.

## Materials and methods

### Cell culture and treatment

K562 (CCL-243, ATCC, Manassas, VA), U937 (CRL-1593.2, ATCC), and HL-60 (CCL-240, ATCC) cells were maintained in RPMI 1640 medium supplemented with 10% (v/v) heat-inactivated fetal bovine serum (FBS) (10099141, Gibco, Auckland, New Zealand). HeLa cell line (CCL-2, ATCC) was cultured in DMEM (10569044, Gibco, USA) supplemented with 10% FBS. Authentication of these cell lines were conducted by short tandem repeat (STR) markers, and no mycoplasma contamination was detected. All cell lines were supplemented with 1% penicillin-streptomycin-glutamine solution (SV30082.01, Hyclone, Utah, USA) and cultured at 37 °C in a humidified atmosphere containing 5% CO_2_. For treatment, K562, U937, or HL-60 cells were seeded at a density of 1 × 10^5^ cells/ml, then cultured for 72 h with 30 µM Hemin (51280, Sigma-Aldrich, Missouri, USA), 2 µM 12-O-Tetradecanoylphorbol 13-acetate (TPA) (S1819, Beyotime, Shanghai, China) or 0.16 µM all-trans-retinoic acid (ATRA) (R2625, Sigma-Aldrich) to induce erythroid, granulocytic, and monocytic differentiation, respectively.

### Antibodies

The following antibodies were used in this study: anti-H3K4me1 (ab8895, Abcam), anti-H3K27ac (ab4729, Abcam), anti-5-methylcytosine (ab10805, Abcam), anti-GATA1 (ab11852, Abcam), anti-TAL1 (C-4) (sc-393287X, Santa Cruz), anti-CEBP/β (ab15050, Abcam), anti-c-Jun (G-4) (sc-74543X, Santa Cruz), anti-PU.1 (B-9) (sc-390659X, Santa Cruz), and anti-Flag (M2) (F1804, Sigma-Aldrich). Anti-mouse IgG HRP-linked antibodies (G-21040, Invitrogen) and anti-rabbit IgG HRP-linked antibodies (G-21234, Invitrogen).

### Plasmid construction for expression of dCas9-effector proteins and gRNAs

The constructs, pcDNA-dCas9^p300 Core^ (61357, Addgene)^38^ pcDNA-dCas9^p300 Core (D1399Y)^ (61358, Addgene)^38^, pdCas9^DNMT3A^ (71666, Addgene)^39^, and pdCas9^DNMT3A (ANV)^ (71685, Addgene)^39^ were from Addgene. gRNAs targeting the *MYB* promoter and enhancer regions were designed using Feng Zhang lab’s Target Finder software (http://crispr.mit.edu). Best guides, with highest score of the inversed likelihood of off-target binding, were selected, and the gRNA sequences are shown in supplementary Table 1. Expression plasmids for gRNAs were constructed by cloning annealed oligos into pSPgRNA (#47108 Addgene)^54^ using BbsI (R0539, NEB) and T4 ligase (M0202, NEB). Then these plasmids were transfected into K562 cells using Lipofectamine 3000 (L3000015, Invitrogen) according to the manufacturer’s instructions. Forty-eight hours later, cells were harvested for analysis.

### Lentivirus production and cell transduction

For creation of the shGATA1 vectors, oligonucleotides for shGATA1 (sequences are shown in supplementary Table 1) were annealed and ligated into the digested pLKO.1-puro vector at the EcoR I and AgeI sites. For creation of the GATA1 overexpression vector, GATA1 PCR product was cloned into pLVX-IRES-NEO at the EcoRI and XbaI sites. In all, packaging plasmids pCMV-VSVG, pCMV-DR8.91 and the relevant lentiviral transfer vectors in a 3:8:10 mass ratio were cotransfected into 293T cells using TurboFect Transfection Reagent (R0531, ThermoFisher). The media containing lentivirus particles were collected after 48 h. And used to infect K562 cells immediately in the presence of 8 μg/ml hexadimethrine bromide (H9268, Sigma-Aldrich). Cells were collected for analysis after 72 h.

### Quantitative real-time PCR analysis

Total RNA was isolated using TRIzol reagent (15596-018, Invitrogen). And 1 μg of total RNA was reverse-transcribed with PrimeScript™ RT reagent Kit with gDNA Eraser (RR047A, TaKaRa, Beijing, China). The levels of specific RNAs were measured using a Light Cycler 480II real-time PCR machine and the iTaq™ Universal SYBR Green Supermix (1725124, Bio-Rad, Hercules, CA, USA) according to the manufacturer instructions. All samples were assayed in triplicate. Data were normalized to a human *Gapdh* (glyceraldehyde-3-phosphate dehydrogenase) control. Relative quantitation was carried out by the comparative threshold cycle (CT) method. Statistical analysis was performed using the GraphPad Prism 8 software. The primer sequences are listed in supplementary Table 1.

### Western blot analysis

Western blot analysis was performed as previously described^25^. Proteins were isolated from cells and protein concentration was determined by a bicinchoninic acid (BCA) assay kit (P0012, Beyotime). Equal amounts of proteins (30 μg) were electrophoresed in 8% SDS-polyacrylamide gels and transferred to nitrocellulose. The membrane was blocked in PBS containing 5% milk powder and 0.1% Tween 20 and incubated at 4 °C overnight with primary antibody and for 1 h at 25 °C with horseradish peroxidase-conjugated secondary antibody. Antibody binding was visualized using Clarity Western ECL Substrate (1705060, Bio-Rad).

### Chromatin immunoprecipitation (ChIP)

ChIP experiments were performed as previously described^24^. In brief, 1 × 10^7^ cells were fixed in 1% formaldehyde for 10 min at room temperature and sonicated to shear the chromatin. Immunoprecipitation of crosslinked chromatin was performed overnight at 4 °C with antibodies. An equal amount of isotype immunoglobulin G (IgG) was used as background control. Primers for ChIP-qPCR are shown in supplementary Table 1.

### Circularized chromosome conformation capture (4 C) assay

4C assay was performed as previously described with minor modification^25^. In brief, 5 × 10^6^ cells were cross-linked by 1% formaldehyde for 10 min at RT, and 0.125 M glycine was added to prevent further cross-linking. Cells were collected and washed twice with PBS, then suspended in lysis buffer to disrupt membranes and isolate chromatin. HindIII (400 U) was used for the first digestion at 37 °C overnight with shaking followed by diluted ligations. After precipitation, chromatin was further subjected to a second round of digestions with a 4-base cutter DpnII and ligation. Primers for the *MYB* viewpoint (Forward: 5′-AGTATTAATTTGCCTTGTCC-3′; Reverse: 5′-GCTAATGTTGGATATATTGC-3′) were designed. Inverse polymerase chain reaction (PCR) was carried out to amplify sample libraries. Multiplexed sequencing was performed on the HiSeq2500 platform. 4C-seq data were analyzed visualization via an R package 4C-ker^55^. Reads were mapped to a reduced genome of unique 29 bp sequences flanking HindIII sites in the hg19 genome.

### Dual-luciferase reporter assay

The *MYB* promoter (chr6:135 501 805–135 502 522, hg19) was amplified, digested with XhoI and BglII and cloned into the pGL4 luciferase reporter vector (Promega). The upstream regions −34ka (chr6:135 467 317-135 468 351, hg19), −34kb (chr6:135 468 624-135 469 679, hg19), −53k (chr6:135 448 094-135 448 922, hg19) and -88k (chr6:135 414 242-135 415 630, hg19) were amplified and cloned into pGL4.10-*MYB*-promoter mentioned above via KpnI/NheI digestion. Then these reporter vectors were transfected into K562 or HeLa cells using Lipofectamine 3000 (L3000015, Invitrogen) according to the manufacturer’s instructions. Luciferase activity was measured using the Dual-Luciferase Reporter Assay System (E1960, Promega) on a FlexStation 3 multi-mode microplate reader. All assays were performed in triplicate and repeated at least three times.

### Bioinformatics and statistical analysis

The ChIP-seq datasets were obtained from the ENCODE project were visualized with the WashU Epigenome Browser (https://epigenomegateway.wustl.edu/)^56^. 4C-seq data were analyzed via the R package 4C-ker (https://github.com/rr1859/R.4Cker)^55^. Statistical significance (*P* < 0.05) for RT-qPCR, ChIP-qPCR, and luciferase reporter assay experiments was assessed by Student’s two-tailed *t*-test. Data were obtained from at least three independent experiments and are expressed as the means ± standard deviation (SD).

## Supplementary information

Supplementary Figure and Table Legends

Supplementary Figure1

Supplementary Figure2

Supplementary Table1

Supplementary Table2

Supplementary Table3

Supplementary Table4

Supplementary Table5

Supplementary Table6

## Data Availability

The data used in this study has been deposited in NCBI’s Gene Expression Omnibus repository and are accessible through GEO accession number GSE140321.

## References

[1] Ramsay RG, Gonda TJ (2008). MYB function in normal and cancer cells. Nat. Rev. Cancer.

[2] Greig KT, Carotta S, Nutt SL (2008). Critical roles for c-Myb in hematopoietic progenitor cells. Semin. Immunol..

[3] Nguyen N (2016). Myb expression is critical for myeloid leukemia development induced by Setbp1 activation. Oncotarget.

[4] Negi V (2017). Hoxa9 and Hoxa10 induce CML myeloid blast crisis development through activation of Myb expression. Oncotarget.

[5] Nakano K, Uchimaru K, Utsunomiya A, Yamaguchi K, Watanabe T (2016). Dysregulation of c-Myb pathway by aberrant expression of proto-oncogene MYB provides the basis for malignancy in adult T-cell leukemia/lymphoma cells. Clin. Cancer Res..

[6] Qu X (2019). c-Myb promotes growth and metastasis of colorectal cancer through c-fos-induced epithelial-mesenchymal transition. Cancer Sci..

[7] Li Y (2016). c-Myb enhances breast cancer invasion and metastasis through the Wnt/beta-Catenin/Axin2 pathway. Cancer Res..

[8] Drier Y (2016). An oncogenic MYB feedback loop drives alternate cell fates in adenoid cystic carcinoma. Nat. Genet..

[9] Zhang J (2013). Whole-genome sequencing identifies genetic alterations in pediatric low-grade gliomas. Nat. Genet..

[10] Clappier E (2007). The C-MYB locus is involved in chromosomal translocation and genomic duplications in human T-cell acute leukemia (T-ALL), the translocation defining a new T-ALL subtype in very young children. Blood.

[11] Lahortiga I (2007). Duplication of the MYB oncogene in T cell acute lymphoblastic leukemia. Nat. Genet..

[12] Tomita A (1998). Truncated c-Myb expression in the human leukemia cell line TK-6. Leukemia.

[13] Frerich CA (2019). N-terminal truncated Myb with new transcriptional activity produced through use of an alternative MYB promoter in salivary gland adenoid cystic carcinoma. Cancers.

[14] Hugo H (2006). Mutations in the MYB intron I regulatory sequence increase transcription in colon cancers. Genes Chromosomes Cancer.

[15] Drabsch Y (2007). Mechanism of and requirement for estrogen-regulated MYB expression in estrogen-receptor-positive breast cancer cells. Proc. Natl Acad. Sci. USA.

[16] Xiao C (2007). MiR-150 controls B cell differentiation by targeting the transcription factor c-Myb. Cell.

[17] Spagnuolo M (2019). Transcriptional activation of the miR-17-92 cluster is involved in the growth-promoting effects of MYB in human Ph-positive leukemia cells. Haematologica.

[18] Bellon T, Perrotti D, Calabretta B (1997). Granulocytic differentiation of normal hematopoietic precursor cells induced by transcription factor PU.1 correlates with negative regulation of the c-myb promoter. Blood.

[19] Hess JL (2006). c-Myb is an essential downstream target for homeobox-mediated transformation of hematopoietic cells. Blood.

[20] Mukai HY (2006). Transgene insertion in proximity to the c-myb gene disrupts erythroid-megakaryocytic lineage bifurcation. Mol. Cell Biol..

[21] Stadhouders R (2012). Dynamic long-range chromatin interactions control Myb proto-oncogene transcription during erythroid development. EMBO J..

[22] Hanlon L (2003). Long-range effects of retroviral insertion on c-myb: overexpression may be obscured by silencing during tumor growth in vitro. J. Virol..

[23] Haviernik P (2002). Linkage on chromosome 10 of several murine retroviral integration loci associated with leukaemia. J. Gen. Virol..

[24] Zhang J, Markus J, Bies J, Paul T, Wolff L (2012). Three murine leukemia virus integration regions within 100 kilobases upstream of c-myb are proximal to the 5’ regulatory region of the gene through DNA looping. J. Virol..

[25] Zhang J (2016). Distal regulation of c-myb expression during IL-6-induced differentiation in murine myeloid progenitor M1 cells. Cell Death Dis..

[26] Stadhouders R (2014). HBS1L-MYB intergenic variants modulate fetal hemoglobin via long-range MYB enhancers. J. Clin. Invest..

[27] Xie S, Armendariz D, Zhou P, Duan J, Hon GC (2019). Global analysis of enhancer targets reveals convergent enhancer-driven regulatory modules. Cell Rep..

[28] Srutova K, Curik N, Burda P, Savvulidi F, Silvestri G, Trotta R (2018). BCR-ABL1 mediated miR-150 downregulation through MYC contributed to myeloid differentiation block and drug resistance in chronic myeloid leukemia. Haematologica.

[29] Ye P, Zhao L, McGirr C, Gonda TJ (2014). MYB down-regulation enhances sensitivity of U937 myeloid leukemia cells to the histone deacetylase inhibitor LBH589 in vitro and in vivo. Cancer Lett..

[30] Buecker C, Wysocka J (2012). Enhancers as information integration hubs in development: lessons from genomics. Trends Genet..

[31] Wang A (2015). Epigenetic priming of enhancers predicts developmental competence of hESC-derived endodermal lineage intermediates. Cell Stem Cell.

[32] Emambokus N (2003). Progression through key stages of haemopoiesis is dependent on distinct threshold levels of c-Myb. EMBO J..

[33] Fuglerud BM (2017). A c-Myb mutant causes deregulated differentiation due to impaired histone binding and abrogated pioneer factor function. Nucleic Acids Res..

[34] Zhao L, Ye P, Gonda TJ (2014). The MYB proto-oncogene suppresses monocytic differentiation of acute myeloid leukemia cells via transcriptional activation of its target gene GFI1. Oncogene.

[35] Li Y (2018). Alterations of specific chromatin conformation affect ATRA-induced leukemia cell differentiation. Cell Death Dis..

[36] Halsey C (2012). The GATA1s isoform is normally down-regulated during terminal haematopoietic differentiation and over-expression leads to failure to repress MYB, CCND2 and SKI during erythroid differentiation of K562 cells. J. Hematol. Oncol..

[37] Rubin AJ (2017). Lineage-specific dynamic and pre-established enhancer-promoter contacts cooperate in terminal differentiation. Nat. Genet..

[38] Hilton IB (2015). Epigenome editing by a CRISPR-Cas9-based acetyltransferase activates genes from promoters and enhancers. Nat. Biotechnol..

[39] Vojta A (2016). Repurposing the CRISPR-Cas9 system for targeted DNA methylation. Nucleic Acids Res..

[40] Liu XS (2016). Editing DNA methylation in the mammalian genome. Cell.

[41] Heintzman ND (2009). Histone modifications at human enhancers reflect global cell-type-specific gene expression. Nature.

[42] van den Heuvel A, Stadhouders R, Andrieu-Soler C, Grosveld F, Soler E (2015). Long-range gene regulation and novel therapeutic applications. Blood.

[43] de Thonel A (2010). HSP27 controls GATA-1 protein level during erythroid cell differentiation. Blood.

[44] Gutierrez L, Caballero N, Fernandez-Calleja L, Karkoulia E, Strouboulis J (2020). Regulation of GATA1 levels in erythropoiesis. IUBMB Life.

[45] Bejjani F, Evanno E, Zibara K, Piechaczyk M, Jariel-Encontre I (1872). The AP-1 transcriptional complex: local switch or remote command?. Biochim Biophys. Acta.

[46] Shinichiro, T. in *Myeloid Leukemia—Basic Mechanisms of Leukemogenesis* 1 edn (eds. Koschmieder, S. & Krug, U.) 239–262 (IntechOpen, 2011).

[47] Jin J (2015). Cooperation of C/EBP family proteins and chromatin remodeling proteins is essential for termination of liver regeneration. Hepatology.

[48] Angeloni A, Bogdanovic O (2019). Enhancer DNA methylation: implications for gene regulation. Essays Biochem..

[49] Shlyueva D, Stampfel G, Stark A (2014). Transcriptional enhancers: from properties to genome-wide predictions. Nat. Rev. Genet..

[50] Luo C, Hajkova P, Ecker JR (2018). Dynamic DNA methylation: In the right place at the right time. Science.

[51] Ordonez R, Martinez-Calle N, Agirre X, Prosper F (2019). DNA methylation of enhancer elements in myeloid neoplasms: think outside the promoters?. Cancers.

[52] Pradeepa MM (2017). Causal role of histone acetylations in enhancer function. Transcription.

[53] Brettingham-Moore KH, Taberlay PC, Holloway AF (2015). Interplay between transcription factors and the epigenome: insight from the role of RUNX1 in leukemia. Front. Immunol..

[54] Perez-Pinera P (2013). RNA-guided gene activation by CRISPR-Cas9-based transcription factors. Nat. Methods.

[55] Raviram R (2016). 4C-ker: a method to reproducibly identify genome-wide interactions captured by 4C-Seq experiments. PLoS Comput. Biol..

[56] Li D, Hsu S, Purushotham D, Sears RL, Wang T (2019). WashU epigenome browser update 2019. Nucleic Acids Res..

