# Supplementary material for: Regulation of MYB by distal enhancer elements in human myeloid leukemia
Source: Cell Death Dis. 2021 Feb 26;12(2):223. doi: 10.1038/s41419-021-03515-z (PMC7910426; doi:10.1038/s41419-021-03515-z)
Supplement: Supplementary file 1 — Supplementary Figure and Table Legends [file 41419_2021_3515_MOESM1_ESM.docx]

**Regulation of *MYB* by distal enhancer elements in human myeloid leukemia**

**Supplementary Figure Legends**

**Supplementary Figure. 1** K562, U937 and HL-60 cells were treated with hemin, TPA and ATRA for erythroid, monocytic and granulocytic differentiation, respectively. Then 4C assay was performed with the *MYB* promoter as viewpoint. Circos diagrams showing the change of intra-chromosomal interactions related to the *MYB* promoter in K562 (A), U937 (B), and HL-60 (C) during differentiation, the distribution plots of normalized read counts were generated by the 4C-ker.

**Supplementary Figure. 2** The enrichment of GATA1 (A), TAL1 (B), CEBP/β (C), c-Jun (D) and PU.1 (E) at promotor, -34kb and -88k regions in K562 cells using ChIP-qPCR for validation of the public ChIP-seq datasets obtained from the ENCODE consortium. Values are represented as percent input normalized by immunoglobulin G control. Data are represented as mean ± SD of three independent experiments, and *P* values are calculated using Student’s t-test (**P* < 0.05; ***P* < 0.01; ****P* < 0.001) in (A), (B), (C), (D) and (E).

**Supplementary Table 1.** Primers, guide RNAs and oligos used in the study.

**Supplementary Table 2.** Potential inter-chromosome interaction loci detected by 4C sequencing in K562, U937, HL-60 and HeLa cells.

**Supplementary Table 3.** Potential inter-chromosome interaction loci detected by 4C sequencing in K562 cells during hemin-induced differentiation.

**Supplementary Table 4.** Potential inter-chromosome interaction loci detected by 4C sequencing in U937 cells during TPA-induced differentiation.

**Supplementary Table 5.** Potential inter-chromosome interaction loci detected by 4C sequencing in HL-60 cells during ATRA-induced differentiation.

**Supplementary Table 6.** Potential intra-chromosome interaction loci detected by 4C sequencing in K562, U937, HL-60 and HeLa cells.
