# Supplementary material for: Regulation of MYB by distal enhancer elements in human myeloid leukemia
Source: Cell Death Dis. 2021 Feb 26;12(2):223. doi: 10.1038/s41419-021-03515-z (PMC7910426; doi:10.1038/s41419-021-03515-z)
Supplement: Supplementary file 4 — Supplementary Table1 [file 41419_2021_3515_MOESM4_ESM.docx]

**Supplementary Table 1: Primers, guide RNAs and oligos used in the study.**

**Real-time PCR primer pair sequences:**

| Gene | Forward primer (5’-3’) | Reverse prime (5’-3’) |
| --- | --- | --- |
| *MYB* | AAGGTCGAACAGGAAGGTTATC | ACTGTTCTTCTGGAAGCTTGT |
| *GATA1* | TGCGGCCTCTATCACAAGATG | CTGCCCGTTTACTGACAATCA |
| *Gapdh* | GGAAGGTGAAGGTCGGAGTCA | GTCATTGATGGCAACAATATCCACT |

**ChIP-qPCR primer sequences for K562 cells:**

| Site | Forward primer (5’-3’) | Reverse prime (5’-3’) |
| --- | --- | --- |
| promoter | GCACAGTTGTAAACCTTGACG | TCCAGCTCCCACTCACT |
| -34ka | AGGAGGCAAGAGAACACATC | GGAGGAGTCTGACTTTGTAGTG |
| -34kb | GATCGTGCTATCCAGAACTAACC | TAAACACTCTCAGGCCCAAAC |
| -53k | CGGTGGTTCACGTCTCTAATC | GAGTCGGTGTTTCTCCATGTT |
| -88k | GGGCAAGTCACTCAATCTCTAA | TATGCCAGGCACTTTGATAGG |

**Guide RNA sequences for CRISPR/dCas9:**

| Gene | Element | Guide | Sequence(5’-3’) |
| --- | --- | --- | --- |
| *MYB* | promoter | gRNA1 | CAAACCCCGCTCCCGGGTCG |
| *MYB* | promoter | gRNA2 | GCTCCCACTCACTGTCGCGG |
| *MYB* | -34k | gRNA1 | TAGTTCTGGATAGCACGATC |
| *MYB* | -34k | gRNA2 | AGTTCTGGATAGCACGATCA |
| *MYB* | -53k | gRNA1 | GAAATTTAGGCCAGGTGCGG |
| *MYB* | -53k | gRNA2 | AGCTAAGCTATGAGGACTTA |
| *MYB* | -88k | gRNA1 | TTTGGACAGGTTGCGTGGAA |
| *MYB* | -88k | gRNA2 | ATCACAGGCGTGACCACACC |

**Sequences for shRNA knockdown:**

| Name | Sequence(5’-3’) | |
| --- | --- | --- |
| *shGATA1* | Forward | CCGGAGCGCCTGATTGTCAGTAAACCTCGAGGTTTACTGACAATCAGGCGCTTTTTTG |
|  | Reverse | AATTCAAAAAAGCGCCTGATTGTCAGTAAACCTCGAGGTTTACTGACAATCAGGCGCT |
| *shNC* | Forward | GATCCUUCUCCGAACGUGUCACGUTTTTCAAGAGAAAACGTGACACGTTAGGAGAATTTTTTG |
|  | Reverse | AATTCAAAAAAUUCUCCGAACGUGUCACGUTTTCTCTTGAAAAACGTGACACGTTAGGAGAAG |
